# A Systematic Review of the Neurocognitive Effects of Psychedelics in Healthy Populations: Implications for Depressive Disorders and Post-Traumatic Stress Disorder

**DOI:** 10.3390/brainsci14030248

**Published:** 2024-03-03

**Authors:** Mario Renato Velit-Salazar, Paulo R. Shiroma, Eloise Cherian

**Affiliations:** 1 Department of Psychiatry and Behavioral Sciences, University of Miami Miller School of Medicine, 1695 NW 7th Ave, Suite 3100, Miami, FL 33136, USA; 2 Mental Health Service Line, Minneapolis VA Medical Center, Minneapolis, MN 55417, USA; 3 Department of Neuroscience, Pomona College, Claremont, CA 91711, USA; eloisecherian@gmail.com

**Keywords:** ayahuasca, drug–psychotherapy combination, lysergic acid diethylamide, MDMA, psilocybin, psychedelics

## Abstract

Objective: This study aims to provide an overview of pharmacological trials that examine the neurocognitive effects of psychedelics among healthy individuals and patients with post-traumatic stress disorder (PTSD) or major depressive disorder (MDD). Methods: The Preferred Reporting Items for Systematic Reviews (PRISMA) was used as a guide to structure and report the findings for this review. A literature search included the MEDLINE database up until December 2022. We included randomized or open-label human studies of MDMA, psilocybin, mescaline, LSD, DMT, or cannabis reporting non-emotionally charged neurocognitive outcomes (“cold cognition”) measured through validated neuropsychological tests. Results: A total of 43 full-text papers on MDMA (15), cannabis (12), LSD (6), psilocybin (9), DMT/ayahuasca (1), and mescaline (0) were included, mostly on healthy subjects. A single article on MDMA’s effects on cognition in subjects with PTSD was included; there were no studies on psychedelics and neurocognition in MDD. Most of the studies on healthy subjects reported detrimental or neutral effects on cognition during the peak effect of psychedelics with a few exceptions (e.g., MDMA improved psychomotor function). Performance on the type of neurocognitive dimension (e.g., attention, memory, executive function, psychomotor) varies by type of psychedelic, dosage, and cognitive testing. Conclusions: Small samples and a lack of uniformed methods across studies preclude unequivocal conclusions on whether psychedelics enhance, decrease, or have no significant effect on cognitive performance. It is foreseen that psychedelics will soon become an available treatment for various psychiatric disorders. The acute and long-term effects on cognition caused by psychedelics should be assessed in future studies.

## 1. Introduction

Psychedelic use and research was documented as early as the late 1800s and has had fluctuating levels of acceptance and stigmatization since then [1]. The “War on Drugs” in the 1970s stunted the growth of psychedelic research; however, with recent efforts to decriminalize psychedelics in many states, their potential for use in psychiatry has become more viable [2]. Psychedelics have been used by many cultures for rituals, recreational, and therapeutic purposes due to their ability to modify cognition, mood, and perceptions [3]. Psychedelics can produce imagery that is clear and vivid or even dream-like, often assisting memory retrieval, or producing strong emotions and insights [4]. Psychedelics have also been described as being able to produce ego-dissolution, which is suggested to be caused by decreased interhemispheric connectivity [5]. These drugs can be classified into classic and non-classic/atypical psychedelics [4]. Classic psychedelics are traditionally labeled as such due to their 5-HT2A receptor agonism, which has been linked to enhanced environmental sensitivity and may enable emotional release [3]. Classic psychedelics include phenethylamines such as mescaline, lysergic acid diethylamide (LSD), psylocibin, and N, N-Dimethyltryptamine (DMT) present in ayahuasca. These psychedelics may additionally produce direct agonism of 5-HT_1A_, 5-HT_2C_, 5-HT_7_, and dopaminergic D_2_ receptors [6]. Atypical psychedelics are a group of unrelated and pharmacologically diverse substances and varied mechanisms [7], which include the dissociative agents such as N-methyl-D-aspartate (NMDA) antagonists such as ketamine and phencyclidine (PCP), empathogens/entactogens such as 3,4-Methylenedioxymethamphetamine (MDMA), and cannabinoids.

Psychedelics have been shown to alter cognition in healthy individuals and could potentially benefit or harm patients with major depressive disorder (MDD) and post-traumatic stress disorder (PTSD). A systematic review of 25 studies [8] involving neuroimaging in healthy controls consuming DMT, psilocybin, LSD, and ayahuasca showed effects on perception and emotion processing, executive functions, complex cognitive functions, and reduced brain activity in key regions of the default mode network, which are involved in mind-wandering and self-awareness. Oral administration of psilocybin and ayahuasca demonstrated consistent excitatory effects in the frontolateral/frontomedial cortex, medial temporal lobe, and amygdala, which are brain regions associated with memory, emotional processing, and introspection [9]. The altered state of consciousness produced by these drugs appeared to disrupt repetitive and pathological patterns of negative thoughts and emotions, commonly observed in mood and anxiety disorders. Psychedelics could, in part, improve these disorders by targeting neural circuits that subserve cognitive processing relevant to executive function and cognitive emotional processing. On the other hand, together with the potential negative emotions that result from psychedelic use such as fear, grief, paranoia, dissociation, and a sense of losing control [10,11], disorganized behavior, distractibility, psychomotor impairment, and visual and auditory alterations have been described [12].

MDD and PTSD are both complex psychopathologies that greatly affect neurocognition [13,14]. The persistent re-experiencing and hyperarousal symptoms following trauma exposure may be related to deficits in inhibition and attentional control that make it more difficult for individuals to disengage from both internal (e.g., emotions, memories) and external stimuli (e.g., triggers) related to trauma exposure [15]. In MDD, decreased concentration, memory deficits, and executive dysfunction are also prevalent [16]. Unfortunately, many of the rapid-acting treatments for MDD, such as electroconvulsive therapy, may cause cognitive deficits as adverse effects [17]. A meta-analysis has also suggested that conventional antidepressants have a negative effect on psychomotor speed and delayed recall in MDD [18].

Given the increasing interest in the therapeutic potential of psychedelics for mental health, this systematic review aims to compile and analyze the existing scientific literature of pharmacological trials that examine the neurocognitive effects of psychedelics among healthy individuals and with patients with PTSD and MDD. Cannabis and MDMA are not traditionally classified as classic hallucinogens; however, they share some pharmacological properties and mechanisms of action with hallucinogenic substances. Endogenous cannabinoids have been found to modulate serotonin, gamma-aminobutyric acid, and glutamate release [19], whereas MDMA releases serotonin from presynaptic 5-HT terminals and is a potent releaser of dopamine and noradrenaline [20]. While there is no consensus upon a classification of cognitive functions, a proposed taxonomy categorizes cognition with emotional valence as “hot cognition” (e.g., anhedonia, negativistic rumination), which differs from “cold cognition” (e.g., learning, memory, executive function, information processing speed, attention/concentration) [21]. Cold cognition is an intellectual and controlled process that requires explicit learning as well as a conscious processing system and functions from a rule-based structure [22]. This review will focus on the dimensions of cold cognition and how it is affected by psychedelic use in these populations.

## 2. Methods

The Preferred Reporting Items for Systematic Reviews (PRISMA) was used as a guide to structure and report the findings for this review, and this study was not registered on Prospero. The literature search included the MEDLINE database up until December 2022. We included (a) studies written in English; (b) randomized or open-label human studies of MDMA, psilocybin, mescaline, LSD, DMT, or cannabis; (c) studies including healthy adults (≥18 years old) or adults with a primary diagnosis of (1) PTSD, or (2) unipolar or bipolar depression according to diagnostic criteria; (d) studies with neurocognitive outcomes measured by validated neuropsychological tests; and (e) studies with a control group with a placebo (active or inactive). We excluded studies (a) conducted among subjects with active psychosis, manic/hypomanic/mixed episodes, or mood disorder induced by medical conditions (e.g., chronic HIV); (b) of psychedelic drugs exclusively in combination with other drugs; (c) investigating cognitive functions with non-standardized experimental paradigms (non-validated neuropsychological tests); (d) with solely emotionally charged cognitive outcomes (“hot cognition”); and (e) considered as case reports.

### 2.1. Information Sources

We searched the MEDLINE database on PubMed for the literature available from inception to 31 December 2022. The MeSH terms for the psychedelics that were included were: “N-Methyl-3,4-methylenedioxyamphetamine”, “Psilocybin”, “Lysergic Acid Diethylamide”, “N, N-Dimethyltryptamine OR Banisteriopsis”, or “Cannabis”.

We created a search query for (1) PTSD, (2) depression, and (3) healthy population. For PTSD, we searched for “Stress Disorders, Post-Traumatic” in combination with each individual psychedelic. For depression, we searched for “Depressive Disorder OR Depression” in combination with each individual psychedelic from our psychedelic list. Lastly, for the healthy population, we searched for “Psychometrics OR Neuropsychological Tests OR Aptitude Tests” in combination with each individual psychedelic.

### 2.2. Search and Trial Selection

**Figure 1:**
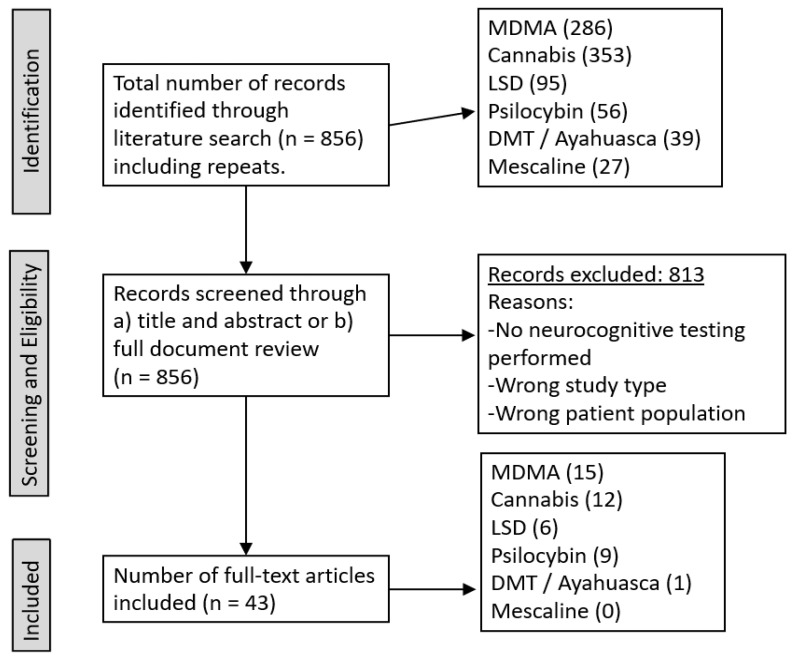
Search and trial selection. Literature search to quantify and determine potential eligibility of results.

The initial literature search to quantify and determine the potential eligibility of results was performed by one of the reviewers (MV-S); the selected articles were then reviewed independently by all three reviewers who determined by consensus which studies were to be included (Figure 1). A final investigator (PS) proofread and handled any arguments. A list of the accepted literature was recorded on Microsoft Excel (Version 4204).

### 2.3. Data Extraction

Data were extracted from included articles after designing a template with parameters in Microsoft Excel. Information pertaining to the author, sample characteristics, design (including time points of administration of drug and effects), neurocognitive and other tests, results, and comments were included (Table 1).

## 3. Results

A total of 856 articles were identified, of which 43 full-text manuscripts were included. MDMA had the largest representation with 15 studies (34%), followed by cannabis with 12 (27%); most excluded studies were related to a lack of standardized neurocognitive testing. In PTSD, a single article on MDMA and neurocognition was included; there were no articles that examined psychedelics and neurocognition in MDD.

### 3.1. Neurocognitive Effects of MDMA in Healthy Population

Population: Fourteen studies published between 2000 and 2017 were included. Study designs were generally crossover, double-blind, placebo-controlled, and randomized. Sample sizes ranged between eight [34] and 42 subjects [31], mostly comprised of men, except for Schmidt et al. and Kuypers et al., which had an almost equal gender distribution. The age ranged between 18 and 39 years. The dosages of oral MDMA were weight-based (between 1.5 mg/kg and 1.7 mg/kg) or fixed (between 75 mg and 125 mg) over the course of one to four separate days. The washout period for crossover studies was at least 5 days. In addition to saline, some studies included active placebos such as methylphenidate (N = 3) [24,28,35], modafinil (N = 1) [24], amphetamine (N = 1) [34], tetrahydrocannabinol (THC) (N = 1), pindolol (N = 2) [41,64], ketanserin (N = 1) [64], and ethanol (N = 4) [20,23,42,65] either alone or in combination with MDMA. The time points to measure immediate neurocognitive performance ranged between 75 and 150 min, which aimed to capture the “expected peak effects” of MDMA. Few studies extended measures beyond 360 min, with one group of investigators reporting the cognitive effects of MDMA during withdrawal phase, namely between 25.5 and 26 h post drug administration [28,35].

Outcomes: The MDMA-induced cognitive effects included attention, response inhibition, memory (i.e., working, visuospatial, semantic), decision making, and psychomotor function.

Attention: The effect of MDMA on attention is unclear. It is possible that specific dimensions of attention (e.g., sustained, selective, divided, and executive) were more susceptible to change. Vollenweider did not detect deficits by MDMA in selective attention as measured by the Stroop test compared to a placebo [30]. However, the same group of investigators using a similar dose of MDMA (1.7 mg/kg) and time point measure (75 min post drug) found a statistical trend of worsening selective and sustained attention during the visual Continuous Performance Test (CPT). The shorted attention span during the Stroop task could have been insensitive to the detection of MDMA-related deficits in contrast to a more complex cognitive task such as CPT, where sustained attention is tested. Lamers found that MDMA improved performance on the Divided Attention task, where psychomotor function is central, but impaired performance in the primary Object Movement Estimation under Divided Attention, a task which depends on response inhibition (executive function). While both tests aimed to examine sustained attention, the effect of MDMA on a subset of associated cognitive dimensions could moderate final attentional outcomes. Finally, four studies failed to elicit any effect by MDMA using the Digit Symbol Substitution Test (DSST) [20,23,34,40,42,46], which measures several other cognitive components including motor speed, attention, and visuoperceptual skills.

Response inhibition: Studies showed that MDMA improved [42], worsened [64], or had a neutral effect [24] on response inhibition. Ramaekers and colleagues found that MDMA improved response inhibition measured by the stop-signal task (i.e., motor impulsivity) but not that by the Iowa gambling task (i.e., cognitive impulsivity). Schmidt et al. reported an increased activation to MDMA in the putative neural network of motor response inhibition during a go/no-go event-related functional magnetic resonance imaging task. Although this study used a similar dose and time measurement as Ramaeker’s study, it failed to replicate behavioral improvement in response inhibition. These discrepant results could be related to differences in study samples (e.g., recreational MDMA users in Ramaekers vs. healthy subjects in Schmidt) and the sensitivity of the motor tasks to elicit changes. In addition, Van Wel found that a single MDMA dose slowed response inhibition, as a measure of motor impulsivity, in both the stop-signal task and the Matching Familiar Figures Test without the worsening of other impulsivity measures.

Memory: Overall, memory is impaired by MDMA [20,28,35,41] but only during the intoxication phase. Kuypers and colleagues demonstrated that both immediate and delayed verbal working memory were impaired during intoxication (30 min post dosing) but not during the withdrawal phase (>24 h post dosing) [28]. Memory impairment was not only attributable to a lesser number of words learned in the immediate recall trials but also to a faster forgetting rate. Dumont and colleagues using 100 mg of MDMA suggested a deficit in the retrieval of verbal information encoded in memory, rather than impairment in the storage of information A second study by Kuypers et al. demonstrated that the impairment during intoxication but not during the withdrawal phase was extended only to simple (i.e., location) but not complex (i.e., location and context) visuospatial memory [28]. Hasler and colleagues using an average higher dose of MDMA (i.e., 1.6 mg/kg) also found an impairment on visuospatial memory during intoxication at 180 min post dosing. Finally, Lamers did not find an acute impairment in semantic memory from a single MDMA dose of 75 mg among healthy recreational users [65].

Psychomotor: The results on psychomotor function vary between a mild improvement and none. Dumont (2008) found no changes at a 100 mg dose, while Lamers (2003) at 75 mg showed increases in psychomotor speed [20,65]. The possibility that the effects of MDMA are biphasic, namely that a low dose of MDMA exhibits more amphetamine-like effects (e.g., arousal, increasing performance), whereas higher doses may elicit more hallucinogenic effects and impair performance [38,44], was supported by the results of two separate studies [34,46] that showed impairment, although mild, in the performance of psychomotor tasks at 125 mg without differential performance between a single [34] and two repeated doses [46]. Interestingly, Dumont (2010) found that 100 mg of MDMA improved psychomotor speed but not accuracy [23].

Decision making: We found two studies that examined the effect of MDMA on decision making in healthy volunteers. Decision making is a higher order executive function that combines anticipation, judgment, reasoning, long-term memory, and working memory. Vollenweider (2005) and colleagues found that MDMA increased response rigidity in a simple two choice prediction but only when there is a high propensity to select a response that generates a correct prediction (i.e., positive reinforcement) [31]. The decision-making patterns induced by MDMA did not change response latency or switching, and this was independent of the psychological state or altered attention to the task. Ramaekers (2006) showed neutral effects during acute intoxication from MDMA on the ability to anticipate and reflect on the consequences of decision making measured in the Iowa gambling task [42].

### 3.2. Neurocognitive Effects of MDMA in PTSD

We found a single study that evaluated the neurocognitive effects of MDMA in a clinical population [26]. Twenty subjects with treatment-resistant PTSD (i.e., a history of failing to respond to selective serotonin and/or serotonin-noradrenergic reuptake inhibitor antidepressants and psychotherapy; Clinician-Administered PTSD Scale for DSM-5 score ≥ 50) were randomized to receive 125 mg of MDMA (plus optional 62.5 mg of two h later) or placebo (i.e., lactose) during an 8 h medication-assisted psychotherapy (MAP) session. A similar second session occurred 3–5 weeks later with an in-between once-a-week, 90 min medication-free therapy integration visit. Neurocognitive domains measured at baseline and two months after the second MAP session included executive function, processing speed, attention, expressive language, metal flexibility, and visual–spatial memory. While the study showed a non-significant change between the MDMA group and the placebo group on major index scores of cognitive tasks, results were limited to show statistical tests. A type II error due to a small sample and a single post-treatment neurocognitive measure at 2 months after the second MAP session may preclude possible detection of any harmful effect of MDMA in PTSD.

#### Summary

Overall, there are not unequivocal findings to conclude that MDMA has deleterious effects across different neurocognitive domains. The most consistent finding is that MDMA at doses between 75 and 100 mg impaired immediate and delayed working, verbal, and visuospatial memory during the acute intoxication phase but not during the withdrawal phase. Interestingly, acute MDMA at 75 or 100 mg improved psychomotor speed with only a mild impairment at a larger dose (125 mg). A similar acute dose–response was elicited in attention with neutral effects at 75 mg and impairment at 1.6 mg/kg (120 mg of MDMA for a subject of 75 kg). Response inhibition was both improved and worsened under 75 mg of acute MDMA. Several studies speculated that these discrepant results, even within the same cognitive dimension (e.g., simple vs. sustained attention) could relate to selective pharmacological manipulation by MDMA on a subset of processes. Neurocognitive outcomes are broad constructs that consist of multiple and interrelated functions and the current behavioral tests may be too insensitive to discern differences among them.

### 3.3. Neurocognitive Effects of Cannabis in Healthy Population

Population: Twelve studies published between 1970 and 2018 were included. Overall, studies were double-blind, placebo-controlled, and randomized. Sample sizes ranged between five [47] and 36 subjects [43], where 67% were male and mostly aged between 20 and 30 years. The dosage of cannabis was either in percent of THC (i.e., 1.8 to 25%), in milligrams (i.e., 2.5 to 60 mg), or was not specified [37]. The washout period for crossover studies was between 24 h and one month. Cannabis was typically inhaled (N = 8) as a single dose per experiment (N = 11). Among the seven studies that compared cannabis to an inactive placebo, four administered oral cannabis. Some studies included an active placebo such as diazepam [39] or cigarettes [47]. One study did not use a placebo [37]. Neurocognition was measured prior to baseline, at baseline, and up to 25 h after drug administration. Most studies (N = 9) had at least one neurocognitive measure within the first h of cannabis administration.

Outcomes: The cannabis-induced cognitive measures included psychomotor skills (e.g., speed, fine motor coordination), attention/concentration, processing speed, memory (e.g., verbal, semantic, working, procedural), and executive function (e.g., cognitive sequencing, response inhibition).

Psychomotor: Out of seven studies, five found worsening motor skills, and two found no effect of cannabis. Cannabis impaired motor ability at 6 mg of inhaled THC measured between 15 and 240 min [40], 10 mg of oral THC measured at two h [48], 20 mg of inhaled THC measured up to six h [39], 10 mg vaporized THC measured up to eight h [47], and an inhaled dose of 3.6% THC measured up to 5.5 h [54]. Other studies reported that psychomotor retardation subsided after 6 h [39] or was none between five and 40 min [51] post cannabis administration. In addition, eye motor function was not significantly impaired with 6 mg of inhaled THC [40] but was impaired with a “high dose” of 3.6% THC [54]. The two studies that showed non-significant psychomotor changes had few measurements (either once or twice) and did not measure beyond 40 min after drug administration. Peak cognitive effects of cannabis occurred between 15 and 60 min and could be seen declining for at least four hours when using the measuring Rotor pursuit task scores [40], which may suggest that psychomotor ability is more likely to deteriorate hours after its use. 

Attention: Cannabis decreased attention in three out of six studies. Decreased attention was found with 10 mg of oral THC measured at one h [50], post-inhaled 3.6% of THC up to 3.5 h [54], and post-vaporized 10 mg and 25 mg of THC up to six h [47]. THC antagonizes the functional connectivity of the dorsal striatum, prefrontal cortex, and hippocampus, which all play a critical role in the processing of salient stimuli, or what we draw attention to [50]. Studies have also suggested there may be an impaired ability to shift attention at least 19 h after abstinence in heavy cannabis users [54]. Studies that assessed attention greater or equal to one h after administration demonstrated conflicting results. Doses as high as 20 mg inhaled [39] or 3.9% inhaled found no significant changes in attention, which contrasts with the deteriorated attention found in other studies at lower doses such as 10 mg oral [50] or 3.6% inhaled [47]. The highest dose of 60 mg vaporized THC [53] was also found to decrease attention. The reasons as to why the lowest and highest doses cause changes in attention is unclear. It is possible that simple attention tasks with shorter durations (e.g., <10 min) may not show changes in attention, but more complex and longer tasks could find impaired performance [39].

Memory: Cannabis decreased memory in four of six studies. Morgan and colleagues [37] found a decrease in semantic memory with an unspecified amount of inhaled cannabis after 24 h; Lane et al. found a decrease in working memory with inhaled 2.2% and 3.9% of THC [47]. Dumont et al. [34] found a decrease in immediate memory with 4 and 6 mg of inhaled cannabis, which was robust after 15 min but diminished 60 min after drug administration (28). Spindle et al. (2018) found a persistent memory impairment up to six h with dose-orderly drug effects of 10 mg and 25 mg of vaporized cannabis [23,47]. Activation of semantic memory may be less efficient in cannabis users as it is possible that semantic memory categories, which represent a variety of knowledge categories, may take longer to activate [37].

Processing speed: Cannabis was shown to decrease processing speed in one of three studies. Spindle et al. (2018) found a decrease in processing speed using the DSST with vaporized cannabis at both 10 mg and 25 mg up to six h [47]. Studies with a neutral effect in processing speed used inhaled THC between 1.8% [54] and up to 8% [51], which suggests that changes in processing speed may be dependent on dose, concentration, and/or method of administration of THC.

Executive function: Cannabis was shown to decrease executive function in one of two studies. Bhattacharyya et al. [43] found a decrease in response inhibition with 10 mg THC oral at one to two h after administration. In the study, the increases in THC showed a non-significant decrease in the connectivity between the striatum and the inferior frontal gyrus on fMRI. The decrease in connectivity suggests a more disinhibited motor function and scattered attention, each of which can deteriorate executive function.

#### Summary

Cannabis exhibited an overall reduction in neurocognitive abilities across studies. A greater dose of inhaled cannabis, which ranged between 6 mg and 20 mg, was associated with lower psychomotor function starting 15 min post administration for up to 6 h. Attention decreased at both low oral doses of 10 mg and high 25 mg vaporized THC without impairment to middle range doses of THC. Routes of administration, and the complexity and duration of tests may explain bimodal changes in attention by THC. In general, relatively low doses of inhaled THC (e.g., 2.2%) caused decreased memory within the first 15 min post administration with greater doses (e.g., 25 mg) impairing memory for up to 6 h [47,52]. Processing speed and executive function had either non-significant changes or worsened performance.

### 3.4. Neurocognitive Effects of LSD in Healthy Population

Population: Six studies published between 1958 and 2022 were included. Overall, studies were double-blind and placebo-controlled. Sample sizes ranged between 16 [57] and 25 [59] subjects; 61% of the total number of subjects were male with an age range between 18 and 61 years. LSD was administered orally, with doses between 5 and 100 µg. Neurocognitive functions were measured at baseline and up to 24 h [58]. Washout periods varied between two days and two weeks. All included studies compared LSD to an inactive placebo.

Outcomes: The LSD-induced cognitive measures included psychomotor function, sustained attention, working memory, executive function (i.e., response inhibition, cognitive flexibility, inhibitory control, perceptual reasoning, decision making, risk-taking behavior), and verbal fluency.

Processing Speed: The only study of LSD and processing speed showed deterioration [60]. Hutten and colleagues administered 5, 10, or 20 µg and measured changes in processing speed at time of administration, and then at two h and four h. Subjects had a decreased speed of information processing (i.e., encoded fewer results on the DSST) with unchanged accuracy even at 20 µg.

Attention: Out of three studies, LSD was shown to improve [60], decrease [57], or have no effect [58] over attention. Hutten and colleagues showed that subjects had fewer attentional lapses on the psychomotor vigilance task with 5 µg and 20 µg between zero and four h after administration. Silverstein [54] found a decrease in attention with 72 µg of LSD measured between 1.5 and 3.5 h after administration; however, the study’s methods are unclear (e.g., randomization/blinding), which preclude more definite conclusions [54]. Wießner and colleagues found no changes in sustained and switching attention when measured 24 h after 50 µg of LSD.

Memory: LSD decreased memory in two studies, had no significant effects in two studies, and improved memory in one study. Memory was unchanged with doses up to 26 µg of LSD at 2.5 h [61], improved with 50 µg of LSD at 24 h [58], and worsened with 72 µg [57] and 100 µg [59] of LSD within 3.5 h post administration. In the study of Wießner and colleagues, LSD enhanced specific aspects of memory including the recall of visuospatial locations, but not auditory–verbal nouns learned before drug administration.

Executive Function: LSD decreased executive function in three of four studies. When administered in doses between 50 and 100 µg [24,58,59], LSD decreased executive function starting from 200 min and up to 24 h after but not at doses less than 50 µg measured up to four h post ingestion [60]. The mechanism may relate to LSD reducing anterior cingulate cortex activation, which plays a role in decision making and error.

Language: LSD improved language in one of one study. Weibner showed an improvement in language with 50 µg of LSD at 24 h. In the study, LSD improved phonological fluency (i.e., ability to produce words that start with a given letter) but not semantic fluency (i.e., ability to generate words in different categories). This suggests that LSD may facilitate frontal-based retrieval strategies such as phonological fluency but not semantic conceptual retrieval, which is temporal-based.

#### Summary

LSD at 50 µg improved language fluency, and memory with lower doses (5 µg and 20 µg) associated with better attention. Larger doses at 72 and 100 µg tended to show lower performance in executive function, working memory, and attention within 1.5 h post administration. Processing speed showed a dose-dependent effect with worsened performance at 5, 10, or 20 µg. In summary, LSD’s neurocognitive effects appear intricately linked to dosage, with lower doses potentially causing minimal effects or a slight improvement, moderate doses generally improving functions, and higher doses leading to cognitive deterioration. The biphasic response observed in attention, with an improvement at lower doses and impairment at higher doses, could be associated with the intricate balance between the stimulation and disruption of serotonin pathways. Similarly, the dose-dependent impact on executive function may be related to the differential engagement of prefrontal cortex regions, where lower doses might enhance certain aspects, while higher doses lead to disturbances in cognitive processes.

### 3.5. Neurocognitive Effects of Psilocybin in Healthy Population

Population: Nine studies were included to examine the neurocognitive effects of psilocybin. All of the studies, conducted between 1996 and 2022, were double-blind, placebo-controlled except for one [56]. Sample sizes ranged between eight [55] and 89 subjects [13], with a nearly equal gender distribution. Subjects were mostly aged between 25 and 39 years, with the majority in their late 20s and early 30s. Psilocybin was most commonly administered through an oral dose and weight-based between 115 μg/kg and 428 μg/kg, except for Rucker et al. [13] which implemented fixed dosages of 10 mg and 25 mg. Three studies compared different doses of psilocybin within the same study [13,66,67]. All studies involved a placebo condition; three studies included ketanserin alone between 40 and 50 mg, and in combination with psilocybin [55,68,69]. Ketanserin was used as a 5-HT_2A_ antagonist to block psilocybin binding and administered 90 min prior to psilocybin. One study included a single high-dose (400 mg/70 kg) condition of the dissociative hallucinogen dextromethorphan [67]. The time points were measured within 420 min post ingestion with expected acute peak effects of psilocybin at 360 min with only one study administering a neurocognitive performance for sustained effects at eight days and 29 days post administration [13].

Outcomes: The psilocybin-induced cognitive effects included attention, processing speed, executive function, memory, response inhibition, and psychomotor function. In addition to cognitive outcomes, results included behavioral changes, mood, and physiological changes.

Executive Functioning: Psilocybin’s impact on executive functioning was examined in four studies with a negative effect in half of them, and a neutral effect in the other half. Barrett and colleagues assessed executive function 2 h after ingestion, with both 20 mg and 30 mg of psilocybin, which demonstrated a significant decrease in the accuracy, but not speed, of substitution recall trials in the DSSTt and attempted responses. This may represent a change in the strategy implemented (i.e., accuracy vs. speed) when using psilocybin but not necessarily an impairment in a neurocognitive function (i.e., executive function). Quednow and colleagues found decreased response inhibition, attentional control, and cognitive flexibility measured by the Stroop test [68]. Based on the contrasting effect of ketanserin, this study suggested that the effect of psilocybin on the performance in the conflict condition of the Stroop test might rather be explained by a dysfunction of conflict monitoring and/or inhibition processes than by an effect on working memory or attention per se. Carter et al. [62], investigated local and global motion processing, which is a predominantly visual attentional task that requires executive function to identify and switch between a superimposed stimulus. They found that at 120 min, global motion processing was significantly reduced in the psilocybin condition compared to the baseline and placebo. Global motion discrimination is believed to be dependent on higher processing areas such as middle temporal area. Ruckers and colleagues used the Cambridge Neuropsychological Test Automated Battery (CANTAB) panel to assess cognitive functioning and, specifically, the spatial working memory strategy to assess executive functioning and planning. Psilocybin at 25 mg showed a trend of better performance at day 29, although there was no difference compared to the placebo condition.

Processing Speed: One study assessed processing speed. Carter and colleagues used the binocular rivalry switch rate to assess the effect of psilocybin at 215 μg/kg every 30 min up until 420 min on attention and processing speed. Psilocybin significantly decreased processing speed, but not accuracy, only at 60 min post ingestion.

Memory: The most common types of memory evaluated were working [13,55,66,67] and episodic [13,67] memory. Psilocybin either worsened [56,66,67] or had a non-significant effect [13,55,66] on memory. Umbricht and colleagues found that psilocybin reduced continuous performance in the context-relevant condition. Psilocybin selectively impairs working memory, specifically, the free recall of words when measured using the letter N-back task [67]; no significant effect on episodic memory by psilocybin was observed. Psilocybin at 250 μg/kg, but not at 115 μg/kg, impaired spatial working memory during peak effects [66]. Conversely, Carter and colleagues found that there was no significant effect on spatial working memory at 120 min after ingestion of 215 μg/kg. No changes in episodic memory compared to the baseline in either psilocybin-dose conditions (10 or 25 mg) were found 29 days after administration [13].

Attention: The effect of psilocybin on attention is unclear and probably related to the differential susceptibility for change depending on the type of attention (e.g., sustained, selective, divided, and executive). Assessed by CANTAB, Rucker [13] concluded that no detrimental effects on cognitive functioning, including attention, were observed. Additionally, Umbricht et al. [57] observed a non-significant difference in attention compared to a placebo when measured at 70 min post ingestion. Quednow and colleagues found that psilocybin increased errors and response time in the interference condition of the Color–Word Stroop test. Carter and colleagues found no reduction in accuracy but an increase in phase duration during a binocular rivalry assessment conducted 70 min after psilocybin administration. Previously, the same investigators found that both the psilocybin condition and increased time had led to impaired attentional tracking abilities when assessed 120 min post ingestion.

Psychomotor: Two studies consistently found impairment across different components of psychomotor skills. Psilocybin significantly altered time perception leading to impaired temporal control of behavior demonstrated through a reduced preferred tapping rate and increased reproduced interval durations [66]. Psilocybin at both 20 mg/70 kg and 30 mg/70 kg showed ad significantly impaired hand–eye coordination 2 and 4 h after ingestion compared to the placebo condition [67]. Balance was also significantly impaired for both psilocybin doses at the same time marks. Interestingly, when examining the average response time during motor praxis tasks, a consistent increase in response time, but not in accuracy, was observed.

#### Summary

Psilocybin exhibited varied effects on neurocognitive functions with weight-based oral doses between 115 μg/kg and 428 μg/kg. Executive function indicated a dose-dependent decrease (20 and 30 mg) in accuracy without affecting speed during substitution recall trials; response inhibition and/or conflict monitoring during the Stroop test at 260 ug/kg (18 mg/70 kg) was also decreased. Processing speed was assessed in one study (215 μg/kg), revealing a significant decrease at 60 min post ingestion without affecting accuracy. Working but not episodic memory was decreased when measured during the peak effect of psilocybin at 0.28 mg/kg (19.6 mg/70 kg). Findings in spatial memory were inconsistent with either a dose-dependent impairment or showing no significant effects. Attention results were inconclusive, with some studies indicating no detrimental effects and others reporting increased errors and response time in certain conditions; effects were observed at various time points, ranging from 70 min to 29 days post ingestion. Psychomotor tasks consistently showed impairment during peak effects, affecting time perception, hand–eye coordination, and balance. The processing speed decline occurred at 60 min post ingestion. Psychomotor impairments were noted at 2 and 4 h after ingestion. Overall, psilocybin’s effects on neurocognitive functions appear to be neutral or impairing, especially in psychomotor tasks during peak drug effects, emphasizing the need for further research to draw conclusive findings, particularly considering potential long-term cognitive impacts.

### 3.6. Neurocognitive Effects of Ayahuasca in Healthy Population

We found a single study that evaluated the neurocognitive effects of ayahuasca in a clinical population [70]. Twenty-four subjects were randomized to receive 100 mg of ayahuasca tea, and had executive function, working memory, and attention measured at the 2 h mark. When compared to healthy controls, ayahuasca worsened working memory and executive function but decreased reaction time. Study limitations include the fact that some of the users were occasional or long-term users of ayahuasca who had stopped use 15 days prior to the study.

## 4. Discussion

This systematic review summarizes the effect of psychedelics on different domains of “cold cognition” (see Table 2). Overall, and except for a single study of MDMA in PTSD subjects, studies were conducted in small samples of a healthy population with greater emphasis to assess cognition during the peak effect of psychedelics (e.g., within 4 h after administration). The different dosages, and various tests for similar neurocognitive domains, preclude definite conclusions from head-to-head comparisons even within the same type of psychedelic; however, most of the studies showed acute detrimental or neutral effects of psychedelics in cognition with a few exceptions. MDMA improved psychomotor function, motor response inhibition, and divided attention (where psychomotor function is central) when measured acutely after a dose of 75 mg. MDMA has an amphetamine-like pharmacological action, which may explain these results. LSD improved visuospatial, but not verbal, memory as well as verbal fluency when measured 24 h after a dosing of 50 μg. Notably, improvement occurred on cognitive material learned before drug administration.

Most of the studies consisted of self-selected men in their 20–30 s, and this is one of the limitations as sex-specific changes in neuroplasticity have been shown to be related to the rapid antidepressant [18] and anti-anhedonic effect [71] of ketamine. Female rats have a lower sensitivity to LSD behavioral actions when 7-β-estradiol and progesterone are at their highest [16]; male, but not female, rats showed increased anxiety behavior directly after prolonged ayahuasca administration [10]. The environment of controlled laboratories also differs from that where people have historically taken psychedelics (e.g., religious ceremonies, “rave” parties) or where patients are currently under study such as MDMA-assisted psychotherapy. Furthermore, specific actions of psychedelics within the same pharmacological class (e.g., classic psychedelics) are highly variable. LSD exhibits affinity for 5-HT_1A/D_, 5-HT_2A/B/C_, and 5-HT_6_, dopamine D_1_ and D_2_, and α-adrenergic receptors, while DMT and its analog 5-MeO-DMT are agonists of 5-HT_1A/D_, 5-HT_2A_, and 5-HT_6_ receptors. The antidepressant effects of ayahuasca may also be produced by its non-psychedelic β-alkaloids harmine, tetrahydroharmine, and harmaline present in the ayahuasca brew, meaning other compounds not involved in serotonin [72].

While this review focused on “cold cognition”, which excluded the psychedelic effects on emotionally charged cognitive performance such as reward learning and risk taking (“hot cognition”), it is likely that their interaction is central to the maintenance of several psychiatric conditions targeted by psychedelic treatment and, therefore, assessments on both cognitive aspects are desirable. Classic psychedelics have shown to increase cognitive flexibility [11], creative thinking [25], and insightfulness [45]; however, there are a scarcity of clinical studies on the effect of psychedelics on “cold cognition” such as memory or attention. It is known that 5-HT_2A_ receptors are widely distributed in the central nervous system, especially in brain regions that are essential for learning and cognition. A consistent post-mortem brain finding in patients with Alzheimer’s disease is a marked reduction in the density of 5-HT_2A_ receptors [49]. The stimulation of 5-HT_2A_ receptors in dorsolateral PFC improved spatial working memory in primates [62]. THC increased the number of connections between brain cells in the hippocampus and reversed age-related cognitive decline in old mice [28]. Similar to the theoretical proposal that psychedelics reduce psychiatric symptoms by opening a critical period of plasticity with exquisite sensitivity to environmental input [63], future studies may examine the neurocognitive effects of psychedelics in combination with cognitive training in psychiatric conditions.

Arguably, psychedelics will become mainstream medications and their use will be expanded among healthy subjects beyond the treatment of psychiatric conditions. The neurocognitive impact of psychedelics is unclear at present. Website initiatives to question the effects of microdosing of psychedelics on cognition using citizen scientists, meaning volunteers who collect and/or process data as part of a scientific enquiry, have been conducted [21]. Future studies should include cognitive performance as a safety measure and as a possible modifier of any clinical outcome including the psychedelic’s mystical experience. Use of valid and reliable standardized testing for long-term outcomes at various medication dosages among a more diverse population will help to overcome some of the limitations already mentioned.

## Figures and Tables

**Table 1:** Summary of studies reporting neurocognitive effects of psychedelics.

**MDMA in PTSD Population**							
**Reference**	**Study Sample**	**Design**	**Drug**	**Neurocognitive Outcomes**	**Other Outcomes**	**Results**	**Limitations**
Mithoefer, 2011, USA [23]	N = 20 (3 men), age 40.4 years. PTSD (war/crime related)	Randomized, double-blind, placebo-controlled.	MDMA 125 mg PO (start) + 62.5 mg PO two hours later vs. placebo (lactose) with concurrent psychotherapy (each session of 8 h). Time point: baseline and 2 months post second session. Washout 3–5 weeks.	Executive function, processing speed, attention, expressive language, mental flexibility, and visual–spatial memory.	PTSD severity, other psychiatric symptoms, and physiological measures.	No significant group differences on any cognitive measure.	Only statistical tests were reported. Type II error is possible due to small sample size; a single post-treatment neurocognitive assessment at 2 months after the second session.
**MDMA in Healthy Population**							
**Reference**	**Study Sample**	**Design**	**Drug**	**Neurocognitive Outcomes**	**Other Outcomes**	**Results**	**Limitations**
Vollenweider, 1998, Switzerland [24]	N = 13 (10 men), mean age: 29 years (range 23–47 years). Use frequency: MDMA-naïve.	Double-blind placebo-controlled.	MDMA (1.7 mg/kg) vs. placebo. Time points: 75 min post drug. Washout 2–4 weeks.	Selective attention.	Mood and consciousness rating scales.	MDMA did not impair selectiveattention as measured by the Stroop test.	Small sample; Stroop test was administered once.
Gamma, 2000, Switzerland [25]	N = 16 (10 men), mean age 26 years. Use frequency: MDMA-naïve.	Double-blind, randomized, crossover, placebo-controlled.	MDMA (1.7 mg/kg) or placebo in 2 separate days. Time point: 75 min. Washout: >2 weeks.	Selective and sustained attention.	Positron emission tomography scans, subjective mood and consciousness rating scales.	MDMA and placebo had non-significant difference in errors in sustained/selective attention test.	Small sample.
Cami, 2000, Spain [26]	N = 8 (8 men), mean age: 26.5 years (range 21–30 years). Use frequency: MDMA on at least five occasions in lifetime.	Double-blind, randomized, placebo-controlled, crossover.	MDMA (75 mg or 125 mg), amphetamine (40 mg), placebo on 4 separate days. Time points: baseline and several times up to 24 h post drug. Washout: ≥1 week.	Psychomotor skills (simple reaction time, attention).	Mood scales, subjective drug sensations scale.	MDMA showed mild decreased psychomotor performance only at 125 mg.	Small population, all men.
Lamers, 2003, the Netherlands [27]	N = 12 (8 men), mean age 23.5 years (range 21–30 years). Use frequency: negative urine testing.	Double-blind, placebo-controlled, three-way crossover, double-dummy.	MDMA (75 mg) vs. ethanol (0.5 g/kg) vs. placebo. Time points: between 1 and 5 h post drug. Washout: 2 weeks.	Psychomotor skills, attention, executive function (planning, semantic memory.)	Toxicological assessments.	MDMA improved psychomotor skills, contradictory results in divided attention, and no effect on executive functions.	Small sample. Tolerance to MDMA may have diminished MDMA-induced effects relative to naive MDMA users.
Farre 2004, Spain [28]	N = 9 (9 men), mean age of 23 years (range 21–33). Use frequency: negative urine testing, no drug use 2 weeks prior.	Randomized, double-blind, crossover, placebo-controlled.	MDMA (100 mg) vs. placebo. Time points: baseline and several times up to 24 h post drug. Washout: 24 h.	Psychomotor skills (simple reaction time, attention.)	Physiological and subjective drug sensations scale.	MDMA showed mild decreased psychomotor performance without difference between interval doses.	Small sample. All men.
Kuypers, 2005, The Netherlands [29]	N = 18 (9 men), mean age: 26.2 years (range 20–39 years). Use frequency: negative urine testing, no drug use 1 week prior.	Double-blind, placebo-controlled, three-way crossover.	MDMA (75 mg) vs. methylphenidate (20 mg) vs. placebo. Time points: 1.5–2 h(intoxication phase) and between 25.5 and 26 h (withdrawal phase) post dosing. Washout: 14 days.	Verbal immediate and delayed working memory, attention.	Sleep scale, mood scale, depression scale.	MDMA impaired immediate and delayed working memory during intoxication but not during withdrawal phase. No difference in attention.	Unclear whether randomization occurred.
Vollenweider, 2005, Switzerland [30]	N = 42 (32 men) mean ages of men and women were 27.0 and 25.4 years, respectively. Use frequency: up to two times in the last 6 months.	Randomized, double-blind, crossover, placebo-controlled.	MDMA (1.5 mg/kg) or placebo. Time points: 120 min. Washout: 2–4 week interval.	Decision making.	Mood and consciousness rating scales.	MDMA affected decision making via a process that is dependent onsuccess or failure.	Methods do not clearly describe blinding of subjects. Psychological state induced by MDMA did not predictthe MDMA-induced decision-making patterns suggesting independent neural systems.
Ramaekers, 2006, The Netherlands [31]	N = 18 (9 men), age range 20–37 years. Use frequency: negative urine testing, no drug use 1 week prior.	Double-blind, placebo-controlled, six-way crossover.	MDMA (75 or 100 mg) or placebo, alone or in combination with ethanol (0.06 g/dL). Time points: 1.5–2 h post drug. Washout: ≥1 week.	Response inhibition, decision making.	Pharmacokinetic assessments.	MDMA improved response inhibition but did not affect decision making. MDMA did not affect alcohol-induced impairment in response inhibition tasks.	Recreational MDMA users. Small sample size.
Kuypers, 2007, The Netherlands [32]	N = 18 (9 men), mean age: 26.2 years (range 20–39 years). Use frequency: negative urine testing, no drug use 1 week prior.	Double-blind, placebo-controlled, three-way crossover.	MDMA (75 mg) vs. methylphenidate (20 mg) vs. placebo. Time points: 1.5–2 h (intoxication phase) and between 25.5 and 26 h (withdrawal phase) post dosing. Washout: 14 days.	Simple (location) and complex (location and context) visuospatial memory.	Pharmacokinetic assessments.	MDMA impaired visuospatial memory of location but not of contextual information and only during intoxication phase.	Unclear whether randomization occurred.
Dumont, 2008, The Netherlands [20]	N = 16 (12 men) mean age: 22.1 years (range 18–29 years). Use frequency: negative urine testing.	Double-blind, randomized, 4-way, crossover, placebo-controlled study.	MDMA (100 mg) PO (or placebo) and an ethanol (10%) (or placebo) infusion on 4 separate days. Time points: 0–90 min. Washout: 7 days.	Reaction time, memory (verbal and visual), psychomotor function, visuospatial/visuomotor function, attention.	Mood rating scales.	MDMA impaired both visual and verbal delayed memory with less consistent impairment in attention. Reaction time, psychomotor, visuospatial/visuomotor functions were not affected.	Limited physical activity and body temperature elevation may not show fully enhanced MDMA effects.
Hasler, 2009, Switzerland [33]	N = 15 (15 men) mean age: 24.3 years (range 20–36 years). Use frequency: seven subjects were drug-naïve, others had single use of MDMA, LSD, and/or psilocybin.	Double-blind, placebo-controlled within-subject design.	MDMA (1.6 mg/kg), pindolol (20 mg), MDMA + pindolol, or placebo, on 4 separate days. Time points: 0–180 min. Washout: min 2 weeks.	Attention, associative learning, visual working memory and planning (executive function).	Assessment of altered states of consciousness and mood states.	MDMA caused decreased sustained attention and impaired visual–spatial working memory.	Small sample size, all male.
Dumont, 2010, The Netherlands [34]	N = 16 (12 men) mean age: 22.1 years (range 18–29 years). Use frequency: negative urine testing.	Four-way, double-blind, randomized, crossover, placebo-controlled.	MDMA (100 mg) PO (or placebo) and an ethanol (10%) (or placebo) infusion on 4 separate days. Time points: 0–360 min. Washout: 7 days.	Psychomotor speed and accuracy, attention.	Postural stability, mood, subjective drug experience and psychedelic effects.	MDMA increased psychomotor speed but not accuracy.	Relevance of the effects measured for actual driving performance is debatable.
Van Wel 2012, The Netherlands [35]	N = 17 (9 men), mean age: 22.7 years (range 19–27). Use frequency: negative urine testing. Mean of 10.9 times MDMA use in the previous year.	Double-blind, placebo-controlled, within-subject design.	Pretreatment (ketanserin vs. pindolol vs. placebo) + treatment (MDMA 75 mg vs. placebo). Time point: 1.5 h. Washout: min 7 days.	Impulsivity/response inhibition.	Mood states.	MDMA slows inhibitory (motor) and reflective (cognitive) response.	Unclear effects of MDMA on impulsivity probably related to tasks that measure multiple neurocognitive processes.
Schmidt, 2017, Switzerland [36]	N = 21 (10 men), age range 21–30 years. Use frequency: negative urine testing, less than 5 times drug use (except THC) within last 2 months.	Double-blind, randomized, placebo-controlled, crossover design.	MPH (60 mg), modafinil (600 mg), MDMA 125 mg, and placebo on 4 separate days. Time points: 75 and 150 min. Washout: 7 days.	Response inhibition.	Psychometric assessment, fMRI brain activation mapping.	MDMA did not improve inhibitory performance (despite neural changes) compared to placebo.	The decreased number of inhibition trials limits the functional relevance of the behavioral results.
**Cannabis in Healthy Population**							
**Reference**	**Study Characteristics**	**Design**	**Drug**	**Neurocognitive Outcomes**	**Other Outcomes**	**Results**	**Limitations**
Wallace, 2007, USA [37]	N = 19 (11 men), mean age 29 years. Use frequency: no drug use 1 month prior.	Double-blind, randomized, placebo-controlled, crossover design.	Cannabis (2%, 4%, 8% THC) or placebo. Inhaled. Time points: 5 and 40 min. Washout: 1 week.	Psychomotor speed, attention, processing speed.	Neurosensory testing, vital signs, subjective intoxication, pain scores to capsaicin injection, Beck depression inventory.	There were no significant changes in neurocognitive outcomes vs. placebo.	Of the 19 subjects, only 15 finished the protocol.
Kaufmann, 2010, Austria [38]	N = 15 (0 men) age range 19–29 years. Use frequency: negative urine test.	Double-blind, randomized, active placebo-controlled, 2-way crossover study.	Cannabis (20 mg THC) or diazepam (5 mg). Inhaled. Time points: 0 h, 3 h, 6 h. Washout: 4 weeks.	Attention, concentration, psychomotor skills.	Brief psychiatric rating scale, subjective feelings (tiredness, “feeling high”) scale.	Cannabis caused decrease in psychomotor skills at 3 h post ingestion.	Study suggests diazepam 5 mg is not equivalent to cannabis 20 mg THC.
Bhattacharyya, 2015, UK [39]	N = 15 (15 men) age 26.7 (±5.7) years. Use frequency: <15 lifetime uses.	Double-blind, randomized, placebo-controlled, repeated-measures, within-subject design.	Cannabis (10 mg THC), CBD (600 mg) and placebo. PO. Time point: 1 h. Washout: 1 month.	Attention.	fMRI and connectivity, psychopathology rating scale.	Cannabis reduced attention compared to placebo.	Only acute effects of cannabis were measured.
Morgan, 2010, UK [40]	N = 36 (21 men) age 26 (±11) years. Use frequency: negative urine test.	Open-label clinical trial.	Cannabis (unspecified amount) or abstinent condition. Inhaled. Time point: 15 min. Washout: 24 h.	Semantic memory (through semantic priming)	Psychotomimetic state scale, schizotypy trait assessment, subjective effects scale, anxiety/depression scale.	Cannabis decreased semantic memory after 24 h.	Open-label, unspecified amount of cannabis, unvalidated task, post hoc results.
Dumont, 2011, the Netherlands [41]	N = 16 (12 men) age range 18–27 years. Use frequency: negative urine test, max of two exposures per week for 1 year.	Double-blind, randomized, crossover, placebo-controlled design.	Cannabis (4, 6, and 6 mg THC dosed every 90 min), MDMA (100 mg), or placebo (vapor, capsule). Inhaled. Time points: 15, 60, 105, 120, 150, 240 min. Washout: 7 days.	Psychomotor speed and accuracy, procedural learning memory, verbal memory, working memory.	Postural stability, mood rating scale, subjective drug experience and psychedelic effects scale.	Procedural learning, motor skills, and working memory were reduced with cannabis vs. placebo.	Some subjects had considerablecannabis use (two or more exposures per week), and subjects may have developed tolerance to some of the cognitive effects of cannabis.
Lane, 2005, USA [42]	N = 5 (3 men) age range 21–34 years. Use frequency: negative urine testing, used 2–10 times per month.	Placebo-controlled design.	Cannabis (2.2% THC and 3.9% THC) or placebo (0.0001% THC + active cigarette). Inhaled. Time points: 15, 195, 255 min. Washout: 5 days.	Implicit memory, working memory, attention.	Subjective rating scale, vital signs.	Both low/high THC doses impaired working memory.	Small sample with 2 subjects dropping out. Placebo (nicotine) may be activating.
Fant, 1998, USA [43]	N = 10 (10 men) age range 24–31 years. Use frequency: Less than or equal to 3 joints a week.	Double-blind, force-randomization, placebo-controlled.	Cannabis (1.8% THC and then 3.6% THC) or placebo. Inhaled. Time points: 2× prior to consumption, then 8× after (0.25 h–5.5 h, at 23, 24, and 25 h). Washout 3 days.	Psychomotor skills, attention, executive function, processing speed, working memory.	Subjective, physiologic, and performance measures.	Motor skills and attention were impaired, particularly with high THC group; executive function, processing speed, working memory were not significantly affected.	Subjects may have learned tasks when given low dose cannabis, prior to high dose.
Roser, 2009, Germany [44]	N = 24 (12 men) mean age 27.9 years. Use frequency: negative urine test, no drug use 1 month prior.	Double-blind, placebo-controlled crossover design.	Cannabis (THC/CBD −10 mg/5.4 mg), THC (10 mg), or placebo. PO. Time point: 2 h. Washout: 1 week.	Psychomotor skills.	Handedness questionnaire.	Cannabis (THC alone) reduced psychomotor skills.	Oral cannabis extract may cause variable plasma cannabinoid concentration.
Ashton, 1981, UK [45]	N = 20 (12 men) mean age 23.2 years. Use frequency: once a week or less.	Blinded, randomized, design.	Cannabis (2.5 mg, 8 mg, or 10 mg THC) or placebo. Inhaled. Time point: 15 min. No washout/crossover.	Psychomotor skills (reaction time).	Electroencephalography, visual/auditory evoked responses, autonomic responses, mood rating scales, personality characteristics.	No significant group differences on any cognitive measure.	Study decreased subject number to 12 when comparing with placebo.
Bhattacharyya, 2014, UK [46]	N = 36 (36 men) mean age 25.9 years. Use frequency: negative urine test, no drug use 1 month prior, 25 total lifetime uses.	Double-blind, placebo-controlled, within-subject design, counterbalanced drug administration order.	Cannabis (10 mg) vs. placebo. PO. Time point: 1–2 h (1×). Washout 1 month.	Executive function (response inhibition.)	Anxiety scale, psychosis scale, subjective intoxication scale, blood levels THC.	Cannabis increased errors, reduced response latency, and lowered efficiency of response inhibition.	Subjects were all men.
Spindle, 2018, USA [47]	N = 17 (9 men), mean age 27.3. Use frequency: negative urine test, no drug use 1 month prior.	Double-blind, crossover study, within-individuals.	Cannabis (vaporized vs. smoked THC—0%, 10%, 25%.) Inhaled. Time points: 0 h–8 h (10×). Washout 1 week.	Attention, concentration, processing speed, psychomotor skills, working memory.	Subjective drug effects.	Vaporized cannabis deteriorated processing speed, attention, executive function, psychomotor skills, compared to placebo.	Small sample size.
Tinklenberg, 1970, USA [48]	N = 8 (8 men), mean age “in their 20’s”. Use frequency: less than or equal to once a month.	Placebo-controlled.	Cannabis (20, 40, 60 mg) vs. placebo. PO. Time points: 1.5 h, 3.5 h, 5.5 h. Washout 1 week.	Working memory.	None.	Working memory was impaired at 1.5 and 3.5 h in all THC groups.	Small sample size.
**LSD in Healthy Population**							
**Reference**	**Study Characteristics**	**Design**	**Drug**	**Neurocognitive Outcomes**	**Other Outcomes**	**Results**	**Limitations**
Schmidt, 2017, Switzerland [49]	N = 18 (9 men) age range 25–58. Use frequency: no drug use 2 months prior.	Double-blind, randomized, placebo-controlled, crossover study.	LSD (100 µg) or placebo. PO. Time point: 200 min. Washout 1 week.	Executive function (response inhibition.)	Altered states of consciousness, fMRI.	Impaired executive function.	Only a modest number of No-Go trials, blinding was difficult to maintain due to drug effects of LSD.
Wießner, 2022, Brazil [50]	N = 24 (16 men) age range 25–61. Use frequency: no drug use 2 weeks prior.	Double-blind, randomized, placebo-controlled, crossover study.	LSD (50 µg) or placebo. PO. Time points: 0 h, 24 h. Washout 2 weeks.	Memory, executive function (cognitive flexibility, inhibitory control, perceptual reasoning), language (verbal fluency), attention.	Only neurocognitive outcomes measured.	Improved memory and language but impaired executive function.	Possible type I error due to lack of correction for multiple comparison; possible practice effect in subsequent sessions.
Pokorny, 2020, Switzerland [51]	N = 25 (17 men) mean age 25.2 years. Use frequency: no drug use 2 weeks prior.	Double-blind, randomized, placebo-controlled, within-subject design.	LSD 100 µg vs. LSD + ketanserin 40 mg vs. placebo. PO. Time points: 220 min. Washout 2 weeks.	Executive function (decision making, risk-taking behavior), spatial working memory.	Altered states of consciousness.	LSD impaired working memory, and partially affected executive function (cognitive flexibility was affected, but not decision making nor risk taking.)	Small sample size.
Bershad, 2019, USA [52]	N = 20 (8 men), age range 18–40 years. Use frequency: no cannabis use 1 week prior, no other drug use 2 days prior.	Double-blind, placebo-controlled.	LSD (6.5, 13, or 26 µg liquid) vs. placebo. PO. Time point: 2.5 h. Washout 1 week.	Working memory	Drug effect scale, mood scales, physiological effects, altered consciousness scale, simulated social exclusion, convergent thinking.	No significant group differences on any cognitive measure.	Small sample size.
Hutten, 2020, USA [53]	N = 24 (12 men), mean age 22.8 years. Use frequency: no drug use 3 months prior.	Double-blind, placebo-controlled, within-subject design.	LSD (5, 10, or 20 mcg) vs. placebo. Time point: 0 h, 2 h, and 4 h. Washout 5 days.	Sustained attention, processing speed, working memory, executive function.	Emotional processing, drug effect scale, mood scales, physiological effects, altered consciousness scale.	LSD reduced the speed of information processing in the Digit Symbol test, and improved attention. No other significant group differences on other cognitive measures.	Study also examined inter-individual variability and concluded that low doses of LSD have beneficial effects on mood and cognition with increased anxiety (based on individual observation).
Silverstein, 1958, USA [54]	N = 16 (16 men) age range 20–24 years. Use frequency: not specified.	Placebo-controlled.	LSD (72 µg) vs. placebo. Washout: 2 days. Time point: 1.5–3.5 h (1×).	Working memory, attention.	Only neurocognitive outcomes measured.	LSD decreased working memory and attention.	Unclear if randomized or blinded study.
**Ayahuasca in Healthy Population**							
**Reference**	**Study Characteristics**	**Design**	**Drug**	**Neurocognitive Outcomes**	**Secondary Outcomes**	**Results**	**Limitations**
Bouso, 2013, Spain [55]	N = 24 (12 men) mean age range 40.5–51. Use frequency: negative urine testing, no drug use 15 days prior.	Open-label, control group.	Ayahuasca (100 mL tea.) PO. Measured at 2 h after ingestion. No washout period.	Executive function, working memory, attention.	Subjective intensity rating.	Ayahuasca worsened working memory but decreased reaction time (increased attention). Executive function worsened (planning, inhibition, impulsivity.)	Study includes occasional and long term users. Possible learning effects from repeat testing.
**Psilocybin in Healthy Population**							
**Reference**	**Study Characteristics**	**Design**	**Drug**	**Neurocognitive Outcomes**	**Secondary Outcomes**	**Results**	**Limitations**
Carter, 2007, Australia [56]	N = 10 (6 men), mean age 26 years. Use frequency: half of the subjects were psilocybin-naïve, other half reported prior experience. No urine testing.	Double-blind, placebo-controlled.	Psilocybin (215 μg/kg) vs. ketanserin (50 mg) vs. psilocybin (215 μg/kg) + ketanserin (50 mg) vs. placebo. Time point: 0 min, 30 min–420 min. Washout: >2 weeks.	Attention, perception, processing speed.	AMRS (different mood states), 5D-ASC (altered state of consciousness.)	Psilocybin decreased attention, decreased processing speed (increased response time), altered perception. Accuracy was not affected.	Small sample size.
Umbricht, 2003, Zurich [57]	N = 18 (10 men) mean age 25.1 years. Use frequency: Not specified.	Single-blind, randomized, placebo-controlled	Psilocybin capsules (0.28 mg/kg) vs. placebo. Time point: 0 h, 70 min. Washout: not specified.	Executive function, working memory, attention.	Modified Mini-Mental State and Brief Psychiatric Rating Scale.	Psilocybin impaired working memory and executive function.	Small sample size.
Wittmann, 2007, Zurich [58]	N = 12 (6 men), mean age 26.8 years. Use frequency: Half of the subjects were psilocybin-naïve.	Double-blind, placebo-controlled, within-subject design.	Psilocybin (115 μg/kg or 250 μg/kg) vs. placebo. Time point: 0 h–360 min. Washout: 2 weeks.	Processing speed, working memory, motor skills.	Altered State of Consciousness rating scale, Adjective Mood Rating Scale.	Psilocybin impaired processing speed, working memory, psychomotor skills in longer intervals, but not short intervals.	Unclear if randomized. Small sample.
Rucker, 2022, UK [13]	N = 89 (48 men), mean age 36.1 years. Use frequency: 56 subjects were psilocybin-naïve.	Double-blind, randomized, placebo-controlled, between-groups study.	Psilocybin (10 mg or 25 mg) vs. placebo, with therapist support. Time point: day 1, day 8, day 29. No washout/crossover.	Episodic memory, executive function, working memory sustained attention.	Vital signs, social cognition scale, emotional processing and empathy scales.	No difference between groups.	Not powered to identify difference between groups; possible practice effects and selection bias. Blinding was not assessed.
Carter, 2005, Switzerland [59]	N = 8 (5 men), mean age 27 years. Use frequency: 3 subjects were psilocybin-naive	Double-blind, placebo-controlled, within-subject, counter balanced.	Psilocybin (215 μg/kg) vs. ketanserin (50 mg) vs. psilocybin + ketanserin vs. placebo. Time points: 0 h, 120 min. Washout: 2 weeks.	Attention, working memory.	Altered states of consciousness scale.	Psilocybin impaired attention when compared to placebo.	Paper could not differentiate if attention vs. impulsivity was affected. It is possible that difficulty was not well matched to the attentional task.
Barrett, 2018, USA [60]	N = 20 (9 men), mean age 28.5 years. Use frequency: none of the subjects were hallucinogen-naïve.	Double-blind, placebo-controlled within-subject.	Psilocybin: high (30 mg/70 kg), medium (20 mg/70 kg), and low (10 mg/70 kg), vs. dextromethorphan (400 mg/70 kg) vs. placebo. Time points: 0 h, 2 h, 4 h, 6 h. Washout: 3–28 days (mean was 10 days)	Motor skills, working memory, episodic memory, executive functioning.	Only neurocognitive outcomes measured.	Psilocybin impaired associative learning and working memory (free recall).	Some incomplete data due to an inability to fully complete tasks.
Quednow, 2012, USA [61]	N = 16 (13 men), mean age 29.7 years. Use frequency: 14 subjects were psilocybin-naïve.	Double-blind, randomized, placebo-controlled, counterbalanced.	Psilocybin (260 µg/kg) vs. ketanserin (40 mg) vs. both together vs. placebo. Time points: 60 min, 125 min. Washout: 4 weeks.	Attention, executive function.	Altered state of consciousness scale, startle response.	Psilocybin worsened attention and impaired executive function (increased errors).	Subjects were almost all men.
Carter, 2004, Switzerland [62]	N = 9 (5 men), mean age 27.1 years. Use frequency: 4 subjects were psilocybin-naïve.	Double-blind, placebo-controlled, counterbalanced.	Psilocybin (215 µg/kg) vs. placebo. Time points: 0 h, 120 min. Washout: 2 weeks.	Visual perception: local motion processing (contrast sensitivity) and global motion processing (coherence sensitivity) discrimination.	Only neurocognitive outcomes measured.	Visual perception is partially impaired at 120 min (global motion is impaired, local motion is unaffected.)	Small sample size.
Spitzer, 1996, Germany [63]	N = 8 (8 men), mean age 39.4 years. Use frequency: not specified.	Double-blind, placebo-controlled, counterbalanced.	Psilocybin (0.2 mg/kg) vs. placebo. Time points: −60 min, 0 h, +50 min, +150 min, +220 min. Washout: 1 week.	Semantic memory, reaction time.	Only neurocognitive outcomes measured	Psilocybin reduced reaction time in all semantic conditions. Semantic memory was not impaired.	Small sample size.

**Table 2:** Summary of neurocognitive changes with psychedelics.

**PTSD**	**Psychomotor**	**Attention**	**Memory**	**Processing Speed**	**Language**	**Executive Function**	**Response Inhibition**	**Decision Making**
MDMA	n/a	●	●	●	●	●	n/a	n/a
**Healthy Population**	**Psychomotor**	**Attention**	**Memory**	**Processing Speed**	**Language**	**Executive Function**	**Response Inhibition**	**Decision Making**
MDMA	●●	●●	●	n/a	n/a	n/a	●●●	●●
Cannabis	●●	●●	●●	●●	n/a	●●	n/a	n/a
LSD	n/a	●●●	●●●	●	●	●●	n/a	n/a
Psilocybin	●	●●	●●	●	n/a	●●	n/a	n/a
Legend:	● Improve	● No effect	● Worsen	n/a: not applicable				
